# Comparative efficacy of a single-session “Empowered Relief” videoconference-delivered group intervention for chronic pain: study protocol for a randomized controlled trial

**DOI:** 10.1186/s13063-021-05303-8

**Published:** 2021-05-22

**Authors:** Maisa S. Ziadni, Steven R. Anderson, Lluvia Gonzalez-Castro, Beth D. Darnall

**Affiliations:** Department of Anesthesiology, Perioperative and Pain Medicine, Division of Pain Medicine, Stanford University School of Medicine, 1070 Arastradero Road, Suite 200, Palo Alto, CA 94304 USA

**Keywords:** Chronic pain, Single-session intervention, Videoconference-delivered, Pain education, Symptom management, Cognitive-behavioral therapy, Treatment, Randomized controlled trial

## Abstract

**Background:**

Chronic pain is naturally aversive and often distressing for patients. Pain coping and self-regulatory skills have been shown to effectively reduce pain-related distress and other symptoms. In this trial, the primary goal is to pilot test the comparative efficacy of a single-session videoconference-delivered group pain education class to a waitlist control among patients with chronic pain.

**Methods:**

Our study is a randomized clinical trial pilot testing the superiority of our 2-h single-session videoconference-delivered group pain education class against a waitlist control. We will enroll 120 adult patients with mixed etiology chronic pain and randomize 1:1 to one of the two study arms. We hypothesize superiority for the pain education class for bolstering pain and symptom management. Team researchers masked to treatment assignment will assess the outcomes up to 3 months post-treatment.

**Discussion:**

This study aims to test the utility of a single-session videoconference-delivered group pain education class to improve self-regulation of pain and pain-related outcomes. Findings from our project have the potential to significantly reduce barriers to effective psychological treatment for pain, optimizing the delivery of increasingly vital online and remote-delivered intervention options.

**Trial registration:**

ClinicalTrials.govNCT04546685. Registered on 04 September 2020.

## Background

Chronic pain is a problem of considerable public health importance that is estimated to impact approximately 100 million Americans and cost society up to $635 billion annually [1]. Chronic pain is particularly challenging to treat given the limited efficacy of existing pharmacological treatments [2–4] and the multisession nature of current psychological treatments, which are difficult to integrate into healthcare settings and thereby perpetuating medical utilization, disparities, and disease. To date, the USA lacks scalable behavioral medicine for pain, underscoring the need for solutions that are accessible, low-cost, and low-burden.

Despite the documented benefits of cognitive behavioral therapy (CBT) for chronic pain [3, 5], it remains limited by multiple obstacles including treatment time, healthcare costs, travel burden, lack of services for patients in remote or rural areas, and lack of available trained therapists and services [6–9]. Given these barriers, as well as the rapidly expanding need for alternatives to face-to-face encounters due to the COVID-19 pandemic [10], there is an urgent need for brief online and remotely delivered psychological interventions. Online and remotely delivered interventions have been demonstrated to be effective in treating a variety of physical and mental health issues, with Internet-based CBT classified as a well-established treatment for depression, panic disorder, and social phobia [11]. Online CBT has also been found to be effective in treating psychological issues related to living with chronic illnesses, including irritable bowel syndrome, tinnitus, and chronic pain [12], with small-to-moderate reported effect sizes (*d* range 0.04 to 1.23, mean 0.60) in chronic pain [11]. Internet- and remote-delivered CBT is also effective in reducing pain catastrophizing and improving patient attitudes toward chronic pain [13]. Other online interventions for patients with pain include the use of compassionate mind training [14], social media-based online community intervention [15], pain self-management [16], and online hypnosis [17]. Although showing promising results, these were multisession interventions ranging from 20 days to 12 weeks, which can be costly, time-consuming, and burdensome for patients.

Increasingly, the field is trending toward a population health approach that uses ultra-brief and targeted behavioral treatments that have the greatest reach at the lowest cost and burden. Single-session interventions (SSIs) have shown promise and efficacy in diverse populations and health conditions such as serious mental illness [18], anxiety and conduct disorder in youth [19], acute insomnia [20], heavy alcohol consumption in college students [21], trauma and adversity [22, 23], postsurgical pain [24], and general chronic pain [25–29]. Several online SSIs have been conducted as well, with demonstrated feasibility and patient acceptance of online-delivered SSIs for multiple sclerosis pain [30], disordered gambling [31], and adolescent mental health [32–34]. Data suggest that SSIs are only slightly less effective than longer course treatments requiring up to 16 h of treatment time [29, 35]. Effective SSIs, especially if delivered digitally, would eliminate many barriers to care such as lack of available trained therapists, treatment cost and burden, and insurance limits.

Our team has developed and tested a 2-h skills-based SSI (“Empowered Relief”), which was shown to reduce pain-specific distress and improve self-regulation at a 4-week follow-up in a cohort of 57 mixed etiology chronic pain patients receiving treatment at a tertiary referral, multidisciplinary chronic pain clinic [25]. Importantly, in a 3-arm randomized controlled trial, Empowered Relief was non-inferior to an 8-week cognitive behavioral therapy and superior to a health education class for reducing pain-specific distress (i.e., catastrophizing) and improving multiple secondary outcomes at 3 months post-treatment in individuals with chronic low back pain. For the first time, the current study seeks to test the impact of a group-based single-session intervention “Empowered Relief” that is delivered online on bolstering pain and symptom management in adults with mixed etiology chronic pain. Participants who are randomized in the waitlist or usual care do not receive the study intervention and will be instructed to continue with the care they would normally receive as part of their ongoing clinical care. Upon completion of the 3-month study, participants in the waitlist will be invited to enroll in “Empowered Relief.”

## Specific aims

Our specific aim and three corresponding hypotheses are outlined below.

1. We will conduct a randomized controlled trial comparing the preliminary effects of a single-session videoconference-delivered pain education class compared to a waitlist control (i.e., usual care) condition.
  - Hypothesis 1: The single-session intervention (Empowered Relief (ER)) will lead to greater reductions in pain catastrophizing compared to a usual care condition.
  - Hypothesis 2: ER will lead to greater reductions in pain bothersomeness and sleep disturbance compared to a usual care condition.
  - Hypothesis 3: ER will lead to greater reductions in pain intensity, anxiety, depression, and physical function compared to a usual care condition.

## Methods/design

### Overview

**Fig. 1:**
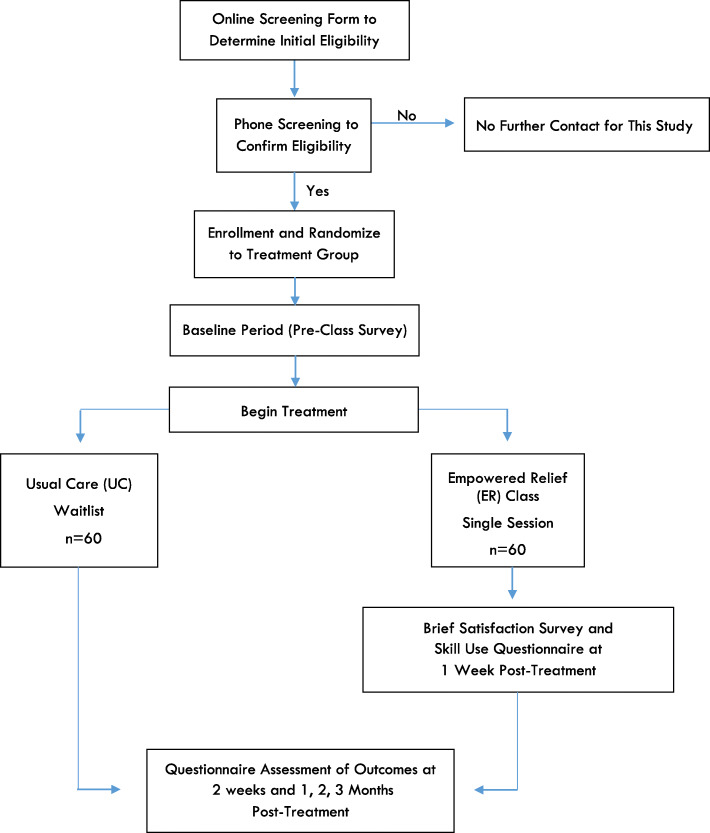
Flowchart of the trial protocol

**Fig. 2:**
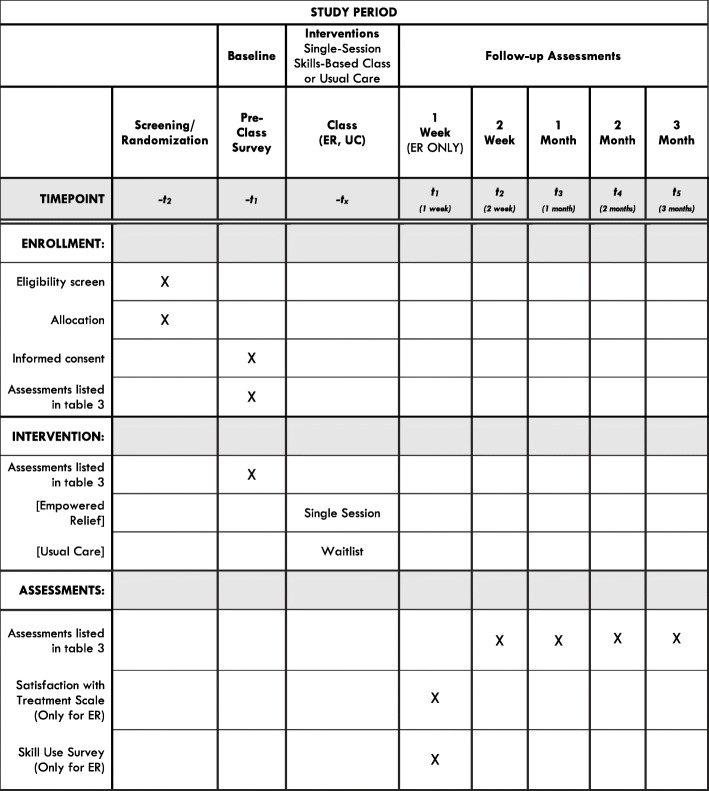
The schedule of enrollment, interventions, and assessments

We are piloting a randomized clinical trial in which individuals with a chronic pain condition are randomly assigned to one of two arms: a single-session videoconference-delivered group pain education class or a waitlist control (WLC) receiving usual care (Figs. 1 and 2). Participants will be followed for 3 months after treatment. Participants will be assessed via an online screening form, a phone screening, enrollment survey, 2-week follow-up, and at 1, 2, and 3 months post-treatment. Team statisticians blinded to participant treatment assignment will assess the outcomes 2 weeks after treatment and after 1, 2, and 3 months. The primary outcome is pain catastrophizing 1 month post-treatment. Secondary outcomes include reductions in pain bothersomeness and PROMIS sleep disturbance at 1 month post-treatment. Tertiary outcomes include reductions in pain intensity, PROMIS anxiety, PROMIS depression, and PROMIS physical function. The protocol for this trial has been approved by the Stanford Institutional Review Board (IRB). All participants will be required to give informed consent to a trained study team member prior to enrollment in the study.

### Study sample and setting

Participants for this trial will be recruited through targeted emails and advertisements at Stanford’s pain management clinics, in addition to the Stanford Systems Neuroscience and Pain Lab (SNAPL) database. All advertisements will direct interested individuals to an online screening form that assesses for initial eligibility. The study will enroll 120 adults (age >18 years) with a chronic non-cancer pain condition and who meet the study criteria (Table 1). The sample size accounts for expected attrition, and eligibility will be assessed by the research staff.

**Table 1:** Inclusion criteria

Inclusion criteria	Rationale	Sources
Pain ≥3 months more than half the time	Study restricted to non-cancer chronic pain	A, TS
English fluency	Ability to complete study procedures	A, TS
Ability to adhere to and complete study protocols		A, TS
Males and females, 18+	Not including children	A, TS

*A*, automated data gathered from REDCap surveys; *TS*, telephone screening

### Inclusion and exclusion criteria

Tables 1 and 2 list the inclusion and exclusion criteria, respectively, as well as the rationale for each criterion and the sources where each criterion will be assessed. Additionally, we require that the participants be willing and available to participate in the full treatment session and able to respond to the enrollment survey (3–6 days before the class date) and post-treatment (2 weeks and 1, 2, and 3 months) follow-up questionnaires.

**Table 2:** Exclusion criteria

Exclusion criteria	Rationale	Sources
Ongoing legal action or disability claim	Source of bias	A, TS
Previous participation in the ER class	Possible bias due to previous exposure to treatment	A, TS
Cognitive impairment, non-English speaking, or factors that would preclude comprehension of material and/or full participation in the study	Not able to complete study procedures	A, TS

*A*, automated data gathered from REDCap surveys; *TS*, telephone screening

### Recruitment procedures

Because the study intervention is group-based and delivered via videoconference, we are recruiting participants in 2–3 large cohorts consisting of 30 participants per class cohort (minimum of 20 participants, maximum of 30 participants per cohort) for both study arms.

Interested individuals deemed initially eligible by the online screening will be further screened over the phone. Eligible individuals will then be invited to enroll in the study and consented with a research staff over the phone, after which they provide an electronic signature to the consent form emailed to them. Participants are randomized following eligibility confirmation and informed consent procedures. Then, participants complete the enrollment survey, which includes information related to their chronic pain and psychosocial well-being.

### Randomization

Enrolled participants will be randomized to one of two study arms: Empowered Relief (ER) and waitlist control (WLC; usual care). An automated program in REDCap will randomly assign a participant to a study arm when enrolled and will ensure blinded randomization, as well as equal numbers in both arms at the end of data collection.

### Blinding

Participants cannot and will not be blinded to the study arm they are randomized to. A clinical psychologist who will be trained in the intervention and has no involvement in the data analysis will deliver the intervention. The study coordinator will be responsible for handling the randomization process through REDCap but will remain blinded to the randomization scheme. All data given to the statistician will be blinded. All research data will be kept separate from identifiers and linked using a participant number. Only the principal investigator will have access to the file linking names and participant numbers, and the file will be stored behind password-protected and fire-walled systems. The team will have access to the final unidentified dataset.

### Study arms

Empowered Relief consists of a single-session 2-h videoconference-delivered group class. After the ER class, participants will receive home-based resources that facilitate ongoing self-regulation and pain self-management. Participants randomized to the waitlist control arm do not receive the study intervention and will be instructed to continue with the medical care they would normally receive. Upon completion of the 3-month study, participants in the waitlist control will be invited to enroll in ER.

#### Single-session videoconference-delivered skills-based pain class (Empowered Relief)

Our group developed the single-session intervention (SSI) for pain management in 2013 with the goal of rapidly equipping patients with skills to self-regulate pain-specific distress. Pilot data revealed significantly reduced pain-specific distress—as indexed by reductions in pain catastrophizing—1 month post-treatment, regardless of comorbid depression and anxiety [25]. The SSI (Empowered Relief) was also the subject of a NIH-funded randomized controlled trial in chronic low back pain [29]; the findings showed that Empowered Relief was non-inferior to an 8-week cognitive behavioral therapy and superior to a health education class for reducing pain catastrophizing and improving multiple secondary outcomes at 3 months post-treatment.

For the present study, for the first time, we seek to pilot test the impact of “Empowered Relief,” a group-based SSI that is delivered online, on bolstering pain and symptom management in adults with mixed etiology chronic pain. A clinical psychologist trained in delivering the 2-h intervention will administer the class via the Zoom platform to groups of enrolled participants. The class is delivered by PowerPoint presentation and includes mind-body pain science, the importance of self-regulation in the context of pain and stress, and evidence-based skills that target pain-specific distress and enhance pain control. Participants will be guided in developing their own self-treatment plan and acquiring the skills necessary to decrease pain and stress-related physiological hyperarousal and to enhance regulation of cognition and emotion within the context of pain. At the end of the class, participants will (1) develop a self-tailored personalized plan to target pain-specific distress, (2) receive a 20-min guided relaxation response audio file, and (3) obtain an electronic copy of the didactic class content. Cohort effects are expected to be minimal due to the single session nature of the class, the highly structured and manualized nature of the class, and the minimal participant interaction via a videoconference platform.

#### Usual care (waitlist control) arm

The control condition will be a usual care comparator, which was selected because clinical trial methodologists [36, 37] argue that waitlist or usual care controls are appropriate for early-phase, proof-of-principle trials where the goal is to promote innovation. Participants in the usual care group will not receive a study intervention and will be instructed by a research study coordinator to continue with the care they would normally receive as part of their ongoing clinical care. Upon completion of all study measures, participants in the usual care condition will be given the opportunity to participate in the intervention arm so as to ensure equality in study participation.

### Class platform

All treatment sessions will occur via the Zoom platform that is password-protected and hosted within the fire-walled Stanford University School of Medicine and Stanford HealthCare systems.

### Instructors

For the ER treatment group, all instructors will be doctoral-level clinical psychologists trained in the treatment of chronic pain.

### Training and monitoring of instructors

ER instructors will be trained in the study protocol for their classes prior to administering treatment. Existing treatment manuals as well as highly structured and standardized class content will assure treatment fidelity. A research coordinator, serving as a fidelity rater, will directly observe the ER classes. Cohort effects are likely to be minimal due to the single-session format and relatively minimal participant interaction.

### Measures

Demographic data, pain characteristics, and physical and psychosocial health measures will be collected at enrollment. Pain catastrophizing will be assessed using the Pain Catastrophizing Scale [38]. Pain bothersomeness will be assessed by a single-item question that is commonly used in chronic low back pain research [39, 40]. PROMIS measures will be used to assess sleep disturbance, pain intensity, pain interference, physical function, depression, and anxiety using short forms [41]. Our group has applied the NIH PROMIS measures in multiple nationally funded clinical pain treatment trials and other studies [42–44] (Table 3).

**Table 3:** Baseline and follow-up measures

Measurement	Brief measure description	Baseline Pre-class survey	Follow-up week 1 (ER class only)	Post-treatment Week 2	Post-treatment Months 1, 2, and 3
Demographics	Date of birth, gender, ethnicity, race, education level, household income, employment, and marital status.	x			
Pain Catastrophizing Scale (PCS) [31]	13-item scale assesses the severity of trait pain catastrophizing tendencies on a 5-point scale (0 = “not at all”; 4 = “all the time”); sum scores range from 0 to 52. The PCS has 3 factors (helplessness, magnification, rumination) and has good psychometrics [31]. Higher score reflects more catastrophizing.	x		x	x
PROMIS measures [27]	NIH PROMIS measures will be used to assess multiple variables of interest, including pain intensity, pain interference, physical function, depression, anxiety, sleep disturbance, social isolation, anger, and fatigue. Short form 4- to 8-item measures will be used to minimize participant burden.	x		x	x
Pain bothersomeness	Single-item measure of pain bothersomeness in the past week from “not at all bothersome” to “extremely bothersome.”	x		x	x
COVID-19	4-item if tested negative and 9-item if tested positive for COVID-19. Assess how the coronavirus impacts chronic pain, day-to-day life, depression, and psychological health.	x			x
Satisfaction with treatment and Zoom (ER group only)	ER group will answer a 13-item survey that assesses the class experience and using Zoom (video-conferencing) as it relates to engagement, relevancy, and usefulness.		x		
Skill use (ER group only)	ER group will answer 5 questions assessing the frequency of skills use learned in class over the past week, from “not at all” to “several times a day.”		x		

#### Baseline (pre-treatment) assessment

Three to 6 days pre-treatment, patients will complete an online baseline survey including demographic information, pain condition and characteristics, pain bothersomeness, pain catastrophizing, and the PROMIS measures. Participants will not be asked to repeat demographic information again, but these measures will be identical to those assessed post-treatment.

#### Post-treatment assessment

One week post-treatment, patients in the ER arm will complete a brief questionnaire assessing patient satisfaction with the intervention, in addition to their frequency of skills use and audio file use. At 2 weeks and 1, 2, and 3 months post-treatment, all participants will complete a set of questionnaires identical to those administered at baseline. The primary study endpoint is 1 month post-treatment. Participants may receive up to $50 for study completion.

##### Primary outcome measure

Our primary outcome measure is pain-specific distress as indexed by pain catastrophizing scores at 1 month post-treatment. Pain catastrophizing will be assessed using the Pain Catastrophizing Scale (PCS), and a total score will be generated using the sum of the 13-items of the PCS. The PCS has valid and reliable psychometrics [38].

##### Secondary outcome measures

Our secondary outcome is pain bothersomeness, whereby participants rate their chronic pain bothersomeness during the previous 7 days on a 0 to 10 numeric rating scale anchored by 0 (not at all bothersome) and 10 (extremely bothersome) that is commonly used in CLBP research [39, 40]. PROMIS sleep disturbance will be assessed with the NIH PROMIS sleep disturbance short form.

##### Tertiary outcome measures

NIH PROMIS measures [41] will be administered to assess pain intensity, anxiety, depression, and physical function. These measures have been successfully applied to pain research [45–48].

### Data collection, quality control, and confidentiality

The online assessments completed by participants will be gathered securely in a REDCap database. No questionnaires will be collected on paper. Additionally, the members of the team will be trained to use and complete case report forms (CRFs), how to review them for completeness, and how to maintain participant confidentiality. The patient flow will be reported according to the Consolidated Standards of Reporting Trials (CONSORT) guidelines [49].

### Protection of human participants and assessment of safety

#### Protection of human participants

The Stanford University Institutional Review Board (IRB) has approved this study.

#### Safety monitoring

This trial will not be monitored by an independent Data and Safety Monitoring Board (DSMB). However, two researchers composed of a physician and a clinical psychologist with knowledge in the treatment of chronic pain conditions will oversee the project. In addition, we have a dedicated research regulatory manager who will provide oversight for all regulatory processes. The team will meet twice a year or on an as-needed basis and will make relevant safety decisions regarding reported participant cases.

#### Adverse experiences

The treatments in this study are not anticipated to pose any risks to participants. However, a study coordinator will review enrolled patient records periodically to monitor for adverse events. In the case of an adverse event, an adverse event case report form will be completed. These will be discussed in monthly team meetings and reported to the IRB annually. Serious adverse events will be reported to the Stanford IRB and NIH. The PI and study committee will evaluate all serious adverse events within 24 h after the study team becomes aware of the incident. All study-related adverse events will be included in the annual report to the NIH, and serious adverse events will be reported within 2 weeks.

#### Stopping rules

The trial will be stopped if (1) either treatment intervention is associated with adverse effects that calls into question the safety of the intervention, (2) there is difficulty in study recruitment or retention that will impact the ability to evaluate study endpoints, (3) new information becomes available during the trial that indicates the need to stop the trial, or (4) other unforeseen situations occur that would warrant stopping the study.

### Statistical issues

#### Sample size and detectable differences

We chose our sample size to ensure adequate power to detect treatment effects on the primary outcome (i.e., pain catastrophizing). The project will enroll 120 participants (ages >18 years) with a diagnosis of chronic non-cancer pain (>3 months more than half the time).

To compare the main effect of ER class on pain catastrophizing scores against the WLC condition, we will plan to enroll 120 participants and have 116 completers (58 per group). The proposed sample size accounts for 4% attrition in each study arm. This is lower than the attrition rate we observed in our Empowered Relief trial [29] of 14.9% or of that seen in pain CBT literature of 18–25% [50, 51], but we believe that the digital format and single-session nature of the intervention will be less burdensome and lead to lower attrition rates. We hope to achieve 80% power to detect medium-large treatment effects on the primary outcome (i.e., pain catastrophizing).

### Statistical analyses

#### Primary analyses

We will use an intent-to-treat approach in all analyses (i.e., the assessment of individuals will be analyzed by a randomized group regardless of participation in the intervention). By doing so, we protect against any confounds that arise as a result of subject dropout.

The main effect of ER on pain catastrophizing will be compared against the WLC using a 2-sample *t* test. Our primary endpoint is pain catastrophizing at 1 month post-treatment. We will also compare the proportion of success rate, defined as ≥30% reduction in pain catastrophizing for a clinically significant treatment response [52].

#### Secondary objectives

To test the secondary and tertiary aims that the ER class will have greater reductions in pain bothersomeness, sleep disruption, pain intensity, anxiety, depression, and physical function compared to WLC, our primary endpoint will be considered at 1 month post-treatment, and its within-subject difference from baseline will be calculated. The mean difference in the ER group will be compared against the WLC arm using the two-sample *t* test. Similarly, we will also compare the proportion of success rate, defined as ≥30% reduction in our outcomes for a clinically significant treatment response [52].

## Discussion

In this trial, we will seek to determine whether a group-based single-session intervention (SSI) that is delivered online is an effective treatment option for persons with chronic pain. In 2019, the US Health and Human Services cited “Empowered Relief” as a promising scalable behavioral pain treatment [53], and for the first time, this study aims to test whether a videoconference-delivered version of the class may similarly effectively and efficiently reduce the burden of chronic pain and improve symptom management. Importantly, it addresses the rapidly expanding need for alternatives to face-to-face encounters due to the COVID-19 pandemic. Finally, the study will identify a proportion of patients who achieve a meaningful reduction in a number of pain-related indices in response to this online single-session intervention. This will facilitate the future application of the digital version of the class across a variety of settings, such as in primary care or in pre-surgical populations, and possibly across chronic health conditions with a primary pain complaint.

## Data Availability

Data will be available on ClinicalTrials.gov (NCT04546685).
